# Velocity of Nervous Force

**Published:** 1864-01

**Authors:** 


VELOCITY OF NERVOUS FORCE.—The *Veterinarian* states that by the aid of a chronoscope M. Hirsch has come to the conclusion that nerves transmit their impressions at the rate of 34 metres a second. M. Heinholtz estimates the velocity of 190 feet per second, but his experiments were performed on the motor nerves of a frog, and those M. Hirsch on the sensitive nerves of a man.

